# Supplementary material for: PLOS Biology 2016 Reviewer and Editorial Board Thank You
Source: PLoS Biol. 2017 Mar 20;15(3):e2002409. doi: 10.1371/journal.pbio.2002409 (PMC5358729; doi:10.1371/journal.pbio.2002409)
Supplement: S1 Reviewer List — (PDF) [file pbio.2002409.s001.pdf]

PLOS | BIOLOGY

PLOS Biology would like to thank all those who reviewed on behalf of the journal in 2016:

Asa Abeliovich
Andrea Ablasser
Helen Abud
Aimee Adam
Carolyn Adler
Deepa Agashe
Merav Ahissar
Joshua Akey
Michael Alfaro
Juan Alfonzo
Nicole Allen
Heike Allgayer
Eric Alm
Luís Amaral
Enrique Amaya
Roberto Amici
Angelika Amon
Hubert Amrein
Madhur Anand
Marc Ancrenaz
Dan Andersson
Amy Andreotti
Ehsan Arabzadeh
Zolt Arany
Jeff Ardron
Luc H. Arnal
Maxim Artyomov
Peter Ashcroft
Kevin Ashley
Etienne Audinat
Johan Auwerx
Narayan Avadhani
Bruno Averbeck
Nikolai Axmacher
Don Ayer
Rava Azeredo da Silveira
Dominik Bach
Tom Baden
Michel Bagnat
Joanna Baker
William Balch
Nabeel Bardeesy
Jose Bargas
Scott Barolo
Rowan Barrett
Douglas Barrick
Alessandro Bartolomucci
Nick Barton
Susan Baserga
Joseph Bass
Alex Bateman
Philip Batterham
Michel Baudry
David Baulcombe
Mark Baxter
Georgii Bazykin
James Bear
Robert Beardmore
L. Beaudrot
Dan Bebber
David Begun
T. Belgard
James Bell
Pedro Beltrao
Suliann Ben Hamed
Dror Ben-Ami
Michael Benton
Andrea Benucci
Carl Bergstrom
Joshua Berke
Ben Berkhout
Ralph Bertram
Debashish Bhattacharya
Ginestra Bianconi
Mariann Bienz
Niels Birbaumer
Ewan Birney
Douglas Bishop
Anthony Bishopp
Jennifer Bizley
Stephen Blacklow
Craig Blackstone
Seth Blair
Laurent Blanchoin
Theodora Bloom
Susan Blum
Justin Blumenstiel
Richard Blythe
Johan Bolhuis
Tobias Bollenbach

Russell Bonduriansky
Timothée Bonnet
Mike Boots
Tilman Borggrefe
Michael Borregaard
Thomas Bosch
Ylan Boureau
Mark Bouton
Rob Boyd
Robert Bradley
Yaniv Brandvain
Thomas Braulke
Dennis Bray
Michael Breakspear
Björn Brembs
Stephane Bressanelli
Roberta Brinton
Nichole Broderick
Micol Bronzini
John Brookfield
Nils Brose
Jurgen Brosius
Stefanie Broszeit
C. Titus Brown
Steven Brown
Robert Brucker
Samantha Brugmann
Michael Brunner
Aurelio Bruno
Jeremy Bruskotter
Mark Brynildsen
Tania Bubela
Ralf Buckley
Alex Bullock
Dirk Bumann
Fred Bunz
Dean Buonomano
Natalie Burden
Steve Burden
Joseph Burger
Mark Burkard
Daniel Burke
David Burr
Nathan Burroughs
Austin Burt
Karen Bush
N. Butterfield
Laura Buttitta
Katherine Button
Danilo Bzdok
Anne Calof
Jane Calvert
Kenneth Cameron
Kerry Campbell
Marco Capogna
Nicholas Carbonetti
Nessa Carey
Jonathan Carlson
Kristian Carlson
John Carr
Jason Carroll
Sean Carroll
Pedro Carvalho
Anne Casper
Marco Catani
Michael Catt
Franco Cauda
Duccio Cavalieri
Maria Chait
Anthony Chalmers
Sumit Chanda
Navdeep Chandel
Chandramouli Chandrasekaran
Karen Chang
Paul Chang
Edwin Chapman
Ian Charest
Trevor Charles
Jonathan Chase
Claire Chazaud
Luonan Chen
Peter Cherepanov
Andrew Chess
Luis-Miguel Chevin
Peter Chien
Nicholas Chiorazzi
Joanna Chiu
Chunaram Choudhary
Pao-Tien Chuang
Jonathan Chubb
Lon Chubiz
Tae-Hwa Chun
Jay Chung
Alberto Ciccia
Andrea Cimarelli
Rafal Ciosk
Damon Clark
Reuben Clements
Miguel Coelho
Gregory Cogan
Frederick Cohan
Gregory Cole

Michael Cole
Hilary Coller
Tom Collett
Anne Collins
Luana Colloca
Paul Conduit
Laura Conforti
Tim Connallon
Andrew Connolly
Joanne Conover
Erik Cook
Mark Cooper
Vanessa Corby-Harris
Tara Cornelisse
Robert Cornell
Ignasi Cos
Christopher Costello
Tim Coulson
Michael Cousin
Stephen Cowen
Charles Cox
Martin Crespi
Clay Cressler
David Croft
Susan Crosthwaite
Nicholas Croucher
Bruce Cumming
Tyler Curiel
Gabriel Curio
Ana Cvejic
Tim Czopka
Fredrick Dahlquist
Shaodong Dai
James Dale
Rebecca Davies
Jenny Davis
Matt Davis
Nathaniel Daw
Ted Dawson
Rob De Boer
Marc de Kamps
Floris de Lange
Gonzalo de Polavieja
Kevin de Queiroz
J. Arjan G. M. de Visser
Nicole De Weerd
Caroline Dean
Dominique Debanne
James DeGregori
Miguel A del Pozo
Kaspar Delhey
Daniele Dell'Orco
Daniela Delneri
Josef Deutscher
Grant Dewson
Mathew Diamond
Michael Dickinson
Susanne Diekelmann
Nai Ding
Ulrich Dirnagl
Nabil Djouder
Chris Doe
Christian Doeller
Raymond Dolan
Richard Dorsky
Angela Douglas
John Drake
Diane Drane
Michael Drew
Karl Drlica
Gabriele Droege
Devin Drown
D. Drummond
Wenzhen Duan
David Dubnau
Rod Dunbar
Mara Duncan
Bénédicte Durand
Monica Dus
Michael Dustin
Paul Dux
Jonathan Dworkin
Fred Dyda
Timothy Ebner
Andrew Edwards
Dylan Edwards
Idan Efroni
Ian Ehrenreich
Gregor Eichele
Howard Eichenbaum
David Eide
Robert Ekblom
Arne Ekstrom
Avigdor Eldar
Charles Eldermire
Florent Elefteriou
Hans Ellegren
Brian Ellis
Stephen Emerson
Ben Emery
Patrick Emery
Richard Emes

Ralf Engbert
Crystal Engineer
Jost Enninga
Kacey Ernst
Cagla Eroglu
Ananias Escalante
Kevin Esvelt
Catherine Etchebest
Jennifer Evans
Adam Eyre-Walker
Oliver Fackler
Damien Fair
Keith Farnsworth
Michael Federle
Richard Fehon
M. Laura Feltri
Martin Fenner
Rafael Fernandez-Chacon
Eberhard Fetz
Charles ffrench-Constant
Greg Field
Barbara Finlay
Adrian Fischer
Alfred Fisher
Brian Fisher
Lila Fishman
Tecumseh Fitch
Thomas Flatt
Elia Formisano
Kevin Foster
Stephanie Fraley
Mirko Francesconi
Steven Frank
Tom Freeman
Michel Frenette
Karl Friston
Robert Froemke
Shigeyoshi Fujisawa
David Funder
Harrison Gabel
Alexander Gail
Jean-Michel Gaillard
Pierre-Henri Gaillard
Karunesh Ganguly
Alexander Gann
Mariano Garcia-Blanco
Andy Gardner
Richard Gardner
Lucia Garrido
Nils Gauthier
Po-Wu Gean
Kerry Geiler-Samerotte
Bruc Gelb
Lisa Genzel
Ulrich Gerland
Samuel Gershman
Timothy Gershon
Fabrizio Ghiselli
Gourisankar Ghosh
Joseph Giacino
Wendy Gilbert
Donna Ginther
Herbert Gintis
Anne-Lise Giraud
Kristina Maria Gjerde
Bojana Gligorijevic
Sebastian Gluth
Jesse Goldberg
Marcia Goldberg
Heather Goldsby
Mark Gomelsky
Jesus Gomez-Gardenes
Zachariah Gompert
Esteban Gonzalez Burchard
Geoffrey Goodhill
Michael Goodisman
Evan Gordon
Vernita Gordon
Jeff Gore
Ankush Gosain
Susan Gottesman
Berthold Gottgens
Jacqueline Gottlieb
Andrew Gracey
Catherine Graham
Melanie Graham
Henry Greely
Michael Greenlief
Brian Gregory
David Gresham
Erwin Grill
Ramon Grima
Ekaterina Grishchuk
Nick Grishin
Jay Groppe
Carol Gross
Helge Großhans
Chiara Guglielmetti
Francois Guillemot
Marc Guitart-Masip
Su Guo
Yan Guo

Robert Guralnick
Ralf Haefner
Melissa Haendel
Ernst Hafen
Stephen Hagen
Matthew Hahn
Oskar Hallatschek
Kosuke Hamaguchi
Wolfgang Hammerschmidt
Iqbal Hamza
William Hanage
Balazs Hangya
William Harcombe
Nicholas Harden
Grahame Hardie
Iswar Hariharan
Stacey Harmer
Mark Harnett
Yasunori Hayashi
David Hayman
Cole Haynes
Sheng He
Stephen Hedges
Christopher Heeschen
Mark Helm
Moritz Helmstaedter
Hugh Hemmings
Andrew Hendry
Marcelo Hermes-Lima
Andreas Herrlich
Franz Herzog
Michael Hickerson
Andres Hidalgo
Caroline Hill
David Hillis
David Hockenbery
Alexander Hoffmann
Eva Hoffmann
Petter Höglund
Marc Holderied
Linda Holland
Gunther Hollopeter
Edward Holmes
Robert Holmgren
Clay Holroyd
Erika Holzbaur
Christopher Honey
Valerie Horsley
Steven Hou
Peter Hraber
Jane Hubbard
Meritxell Huch
Colin Hughes
John Huguenard
Christopher Hunter
Laurence Hurst
John Hutchinson
Niall Hyland
Anthony Hyman
Zoya Ignatova
Chris Illingworth
Shin-ichiro Imai
Beat Imhof
Philip Ingham
Nicholas Ingolia
Dragos Inta
Javier Irazoqui
Manuel Irimia
Harry Ischiropoulos
Masayuki Ishikawa
Matic Ivan
William Ja
Kim Janda
Ralf-Peter Jansen
Daniel Jarosz
Heinrich Jasper
Patrick Jay
Penny Jeggo
Albert Jeltsch
Kim Jensen
Ole Jensen
Torben Jensen
Karim Jerbi
Sebastian Jessberger
Walter Jetz
Lee-Way Jin
Ludger Johannes
Friedrich Johenning
Susan John
Laura Johnston
Kristina Jonas
Russell Jones
Walton Jones
Lucas Joppa
Gabor Juhasz
Suckjoon Jun
Jae Jung
Jon Kaas
Allen Kaasik
Matt Kaeberlein
Marko Kaksonen
Daniel Kalderon

Tobias Kalenscher
Christoph Kaleta
Eric Kalkhoven
Raghu Kalluri
Yukiyasu Kamitani
Harm Kampinga
Wendy Kan
Kunihiko Kaneko
Maya Kansara
Mariusz Karbowski
Katrin Karbstein
Anna Kashina
Purna Kashyap
Itamar Kastner
Vaishali Katju
Aris Katzourakis
Michael Kawaja
Kazutaka Kawatsu
Kendrick Kay
Christoph Kayser
Alex Keene
Darcy Kelley
Jeffery Kelly
Henry Kennedy
Jonathan Kennedy
David Kent
Kinneret Keren
Aaron Kesselheim
Mazen Kheirbek
Chaitan Khosla
Ikuhiro Kida
Michael Kiebler
Clemens Kiecker
Auston Marmaduke Kilpatrick
Trevor Kilpatrick
Haesun Kim
Jeansok Kim
Minsu Kim
Jonathan Kimmelman
Danny Kingsley
Mark Kirkpatrick
Roy Kishony
Istvan Kiss
Axel Kleidon
Jurgen Kleine-Vehn
Jens Kleinjung
Wolfgang Klimesch
Matthew Kloser
Mike Klymkowsky
Sandra Knapp
Dan Knights
Bartha Knoppers
Walter Koenig
Nils Kolling
Alexey Kondrashov
Genevieve Konopka
Benoit Kornmann
Steven Kosak
Sergei Kosakovsky Pond
Christian Kost
Akos Kovacs
Rhett Kovall
Mark Krasnow
Nikolaus Kriegeskorte
Sheldon Krimsky
Richard Kriwacki
Barry Kroll
Elke Krueger
L.E.B. Kruuk
Sergey Kryazhimskiy
Adam Kucharski
Ulrich Kück
Grzegorz Kudla
Rolf Kuemmerli
Natalie Kuldell
Dimitri Kullmann
Inna Kuperstein
Hyungbae Kwon
Tracey-Lea Laba
Thurston Lacalli
Denis Lafontaine
Peter Lakatos
Daniel Lakens
Daniël Lakens
Edmund Lalor
Christophe Lamaze
Angelika Lampert
Karri Lamsa
Nathaniel Landau
Arthur Lander
Michael Landy
Deborah Lannigan
Liana Lareau
Daniel Larremore
Catherine Larrère
Corinne Lasmezas
Michael Lassig
Simon Laughlin
Alison Ledgerwood
Cheng-Yu Lee
Siu Sylvia Lee
Christopher Lefèvre

Ellen Leffler
Ben Lehner
Mate Lengyel
Jonathan Lenoir
David A. Leopold
Cammie Lesser
Sarah Lester
Jun-Yi Leu
Herbert Levine
Joel Levine
Nicholas Levinson
Michael Levy
David Lewis
Kim Lewis
Zachary Lewis
Ian Lewkowich
Loet Leydesdorff
Jianyong Li
Min Li
Thomas Libby
Prisca Liberali
Arthur Liesz
Tom Little
Kent Lloyd
Cecilia Lo
Gerald Loeb
Robbie Loewith
Jan Lohmann
Carlos Lopez-Otin
Rik Lories
Attila Losonczy
Zhenkun Lou
John Loughlin
Ed Louis
Edward Louis
Jan Löwe
Hui Chen Lu
Burkhard Ludewig
Bryan Luikart
Gergely Lukacs
Paul Macdonald
Wendy Macklin
Malcolm Macleod
Luca Magnani
Ryszard Maleszka
Mirjana Maletic-Savatic
Harmit Malik
Julin Maloof
Mark Mandel
Susan Mango
Matthias Mann
Jeremy Manning
Vinicius Maracaja Coutinho
Alberto Marina
Rogier Mars
Dustin Marshall
Alex Martin
Paul Martin
Thomas E. Martin
William Martin
Adam Martiny
Justin Mason
Ruth Massey
Marcello Massimini
John Masters
Seth Masters
Grant Mastick
Juan Mata
John Matthews
Claudio Mauro
Kevin McCann
Susan McCouch
John McCutcheon
Jo McEntyre
Michael Meaney
Paolo Medini
David Meek
Yves Meinard
Christian Meisel
Lucia Melloni
Vinod Menon
Lotfi Merabet
Philipp Mergenthaler
Frederic Mery
Steven Metallo
Martin Michaelis
Daniel Mietchen
Tam Mignot
Irene Miguel-Aliaga
Marco Milan
Samuel Miller
Kathryn Miller-Jensen
John Minna
Christen Mirth
Tim Miyashiro
Yasushi Miyashita
Marek Mlodzik
Andrew Moeller
Jeffrey Mogil
Alex Mogilner
Axel Mogk
Hakimi Mohamed-Ali

Babak Momeni
Denise Monack
Kelly Monk
Scott Montgomery
Arne Mooers
Frits Mooi
Joao Moreira
Ruben Moreno-Bote
Arnaud Moris
Levi Morran
James Morris
Richard Morris
Naomi Morrissette
Christian Mosimann
Hong Moulton
Hugo Mouquet
Joris Mulder
Volker Müller
John Mundy
Bernard Munos
Yasunori Murakami
Coleen Murphy
Geoffrey Murphy
James Murphy
Shona Murphy
Andrew Murray
Risto Naatanen
Paul Nabity
Nicola Nadeau
Matthew Nassar
Dick Nässel
Inke Nathke
Erwin Neher
Matthew Neiditch
Stuart Neil
Maurine Neiman
Jonathan Nelson
Thomas Neufeld
Karla Neugebauer
Anje-Margriet Neutel
Richard Neve
Cameron Neylon
Xavier Nicol
Andreas Nieder
Jens Nielsen
Alex Ninfa
Vivek Nityananda
B. Nixon
Mark Noble
Mohamed Noor
Patrice Nordmann
Ken Norman
Brian Nosek
Peter Novick
Bennett Novitch
Michele Nuijten
Zoltan Nusser
Jon Oatley
Marcel Oberlaender
Jonas Obleser
Michael O'Connor
David Odde
John O'Doherty
Thomas Oertner
Stacey Ogden
Sam Oh
Thomas O'Halloran
Benjamin Ohlstein
Kirsten Oleson
Francisco Oliver
Trudy Oliver
Bjorn Olsen
Stig Omholt
Nikki Osborne
Sarah Otto
Craig Packer
Savita Pahwa
Csaba Pal
Tao Pan
Michael Pankratz
Giuseppe Pantaleo
Hyojin Park
Yungki Park
John Parkinson
Raghuveer Parthasarathy
Anitha Pasupathy
David Paterson
Greg Payne
Marius Peelen
Jonathan Peelle
Zhen-Ming Pei
Stuart Peirson
Lucas Pelkmans
Fanie Pelletier
Rafael Pena-Miller
David Penny
Matjaz Perc
Nathalie Percie du Sert
Alan Perelson
Alex Perkins
Charles Perrings
Mathias Pessiglione

Luiz Pessoa
Townsend Peterson
Chris Petkov
Leonard Petrucelli
Roberto Pezza
Cathie Pfleger
Albert Phillimore
Stefano Piccolo
Hilda Pickett
Ron Pinhasi
Paolo Pinton
J. Pires
Lukasz Piwek
Anne Plant
Matthias Platzer
Steve Polasky
Jessica Polka
Katie Pollard
Martin Polz
Andrew Pomiankowski
Brian Popko
Hugh Possingham
Evan Powers
Jonathan Prather
Jochen Prehn
Jeffrey Price
Elizabeth Pringle
Justin Pritchard
Stephen Proulx
Peter Pryciak
Curt Pueschel
Edward Pugh
Louise Purton
Wenfeng Qian
Nancy Raab-Traub
David Raichlen
Klaus Rajewsky
Santiago Ramirez
Nick Ramsey
David Rand
Afshin Raouf
John Ratcliffe
Kodi Ravichandran
Peter Reddien
Akhilesh Reddy
Rosie Redfield
Roland Regoes
Yvonne Reid
Wolf Reik
Frank Reimann
Tania Reis
Kyu Rhee
Carlos Ribeiro
Vanessa Ribes
Phoebe Rice
Ingmar Riedel-Kruse
Scott Rifkin
Sebastien Rigali
Olivia Rissland
Michael Ristow
Silvio Rizzoli
Silke Robatzek
David Robbe
Edwin Robertson
Lucy Robinson
Chris Rogers
Rajat Rohatgi
Luis Rokeach
Martin Rolfs
Edmund Rolls
Carla Romney
Marian Ros
Michael Rosbash
Susan Rosenberg
Robert Roskoski
Janet Rossant
Ronald Rousseau
François Rouyer
Mathieu Roy
Christian Ruff
Andy Russell
Anil Rustgi
Vanessa Ruta
Jared Rutter
Timothy Ryan
Tim Sackton
Ergun Sahin
Alvaro Sanchez
Alessandro Sancino
Joshua Sanes
Helene Sanfacon
Rafael Sanjuán
George Santangelo
Marco Sardiello
Kai Sassenberg
Cristina Savin
Patricia Scaraffia
Samuel Scarpino
Jorn Scharlemann
Frank Scharnowski
Jürgen Scheller
Philipp Scherer

Reinhold Scherer
Lothar Schermelleh
David Schimel
David Schindel
Andreas Schlosser
Sandra Schmid
Claudia Schmidt-Dannert
Marc Schmidt-Supprian
David Schneider
Felix Schönbrodt
Hinrich Schulenburg
Harald Schulze
Michael Schurr
Claus Schwechheimer
Luca Scorrano
Ian Scott
Stuart Sealfon
Julie Segre
Hank Seifert
Aaron Seitz
Emily Sena
Reza Shadmehr
Orie Shafer
Premal Shah
Michael Sheehan
Noam Shemesh
Kang Shen
Gavin Sherlock
Darryl Shibata
David Shiffman
Oren Shriki
Daniel Shulz
Mark Siegal
Markus Siegel
Bob Siegerink
Eric Siggia
Stephan Sigrist
Alan Silberberg
Gilad Silberberg
Shai Silberberg
Robert Siliciano
Alcino Silva
Elizabeth Silva
Jerry Silver
R. Silver
Miljan Simonovic
Mikael Simons
Roberta Sinatra
Per Jesper Sjostrom
Pontus Skoglund
Inna Slutsky
Gerry Smith
Richard Smith
Evan Snitkin
Michael Snyder
Samuel Sober
Jorge Soberon
Michael Sofroniew
Vikaas Sohal
Anna Sokac
Thierry Soldati
Dieter Söll
Lukas Sommer
Ralf Sommer
Tobias Sommer
Chaoming Song
Hongjun Song
Claudio Soto
Victor Sourjik
Charles Springer
James St John
Eric Stabb
Emmanuel Stamatakis
Radu Stan
Phillip Staniczenko
James Staples
George Stark
Doekele Stavenga
Mitchell Steinschneider
Michael Steiper
Joan Steitz
Philipp Sterzer
Murray Stewart
Rodney Stewart
Walter Stoecker
Peter Stoilov
Zuzana Storchova
Brian Stramer
Matthew Strand
Carly Strasser
Joan Strassmann
Daniel Strech
Eric Strieter
Pierluigi Strippoli
Veit Stuphorn
Joseph Sun
Mark Sundrud
Surachai Supattapone
Karel Svoboda
Charles Swanton
Kenton Swartz
Michael Taborsky

Gentaro Taga
Atsuko Takashima
William Talbot
Elly Tanaka
Mark Tanaka
Andrew Tanentzap
Steven Tannenbaum
Lionel Tarassenko
Ann Tate
Cormac T. Taylor
Guy Tear
Markus Teige
Olivier Tenaillon
Celine Teplitsky
Lenny Teytelman
Luc Teyton
Edward Theriot
Bertrand Thirion
Wesley Thompson
Carl Thummel
Sally Thurston
Gregor Thut
Christian Tidona
Sarah Tishkoff
Bosco Tjan
Sokol Todi
Michel B. Toledano
Seth Tomchik
Susumu Tomita
Frank Tong
Tong Tong
Chris Tonkin
Erdal Toprak
Miguel Torres
Greg Towers
Jan Traas
Elizabeth Tran
Stefan Treue
Nicolas Tricaud
Giorgio Trinchieri
Susannah Tringe
Michael Tsang
George Tseng
Richard Tsien
Naotsugu Tsuchiya
James Turner
Esther Turnhout
Luke Tweedy
Mary Tyler
Hiroki Ueda
Hisashi Umemori
Robert Unckless
Brian Uzzi
Golnaz Vahedi
Pedro Vale
Marcel van Assen
Jeremy Van Cleve
Bart van der Worp
Nees Jan van Eck
Marcel van Gerven
Luc Van Kaer
Joyce van Meurs
Rufin Van Rullen
Wim Vanduffel
Rufin VanRullen
Sonia Vasconcelos
Lidia Vasiljeva
David Vaux
Dimitrios Vavylonis
Jan-Willem Veening
Chris Venditti
Michele Vendruscolo
Kartik Venkatachalam
Nuria Verdaguer
Kenneth Vernick
James Versalovic
Kevin Verstrepen
Pita Verweij
Jonathan Victor
Marco Vignuzzi
Jose Vilar
Mark Viney
Todd Vision
Denis Vivien
Kirill Volynski
Tobias von der Haar
I. Vorechovsky
Anne Voss
Patrik Vuilleumier
Erwin Wagner
David Walker
Neil Walker
David Wallach
Brian Wandell
Angela Wandinger-Ness
David Wang
Jing Wang
Yiwei Wang
Yu-Chiun Wang
Zhao-Qi Wang
Ryan Ward
Jonathan Warner

Martin Warren
Philip Washbourne
Takeo Watanabe
Martin Watterson
Jason Weber
Cornelis Weijer
Leor Weinberger
Michael Weisberg
Michael Weiss
Dominic Wells
Dave Wemmer
Jevin West
Bradley White
Helen White-Cooper
Emma Whitelaw
Andrew Whiten
David Whitney
Søren Wichmann
Hynek Wichterle
Thomas Wight
David Wilcockson
Claus Wilke
Robert Wilkinson
Mark Williamson
Alastair Wilson
Charles Wilson
Robert Wilson
Dennis Winge
Stacey Winham
Alan Wolfe
Christian Wolfrum
Roy Wollman
Jonathan Wolpaw
Mark Woolhouse
Kevin Woollard
Jinhua Wu
Ling-gang Wu
Long-Jun Wu
Karina Xavier
Heng Xu
X.Z. Shawn Xu
Toshihide Yamashita
Yukiko Yamashita
Daniel Yamins
Nan Yan
Wei Yang
Humphrey Yao
Nilay Yapici
Tal Yarkoni
Dag Yasui
Bing Ye
Deborah Yelon
John Yin
Haoqiang Ying
Kevin Young
Eloisa Yuste
Dani Zamir
Marija Zanic
Raz Zarivach
Robert Zatorre
Rolf Zeller
Jianzhi Zhang
Jing Zhang
Xiuren Zhang
Xinyu Zhao
Yi Zhong
Ronghua Zhuge
Daniel Zilberman
Leonard Zon
Antonio Zorzano
