# Supplementary material for: PLOS Biology 2016 Reviewer and Editorial Board Thank You
Source: PLoS Biol. 2017 Mar 20;15(3):e2002409. doi: 10.1371/journal.pbio.2002409 (PMC5358729; doi:10.1371/journal.pbio.2002409)
Supplement: S1 Editor List — (PDF) [file pbio.2002409.s002.pdf]

PLOS Biology would like to thank all those who served on the journal's Editorial Board in 2016:

Anurag Agrawal
Julie Ahringer
Anna Akhmanova
Linda Amos
James Ashe
Alberto Bacci
Nathalie Balaban
Anthony Barnosky
Ben Barres
Nick Barton
Konrad Basler
Renata Basto
Gillian Bates
Peter Becker
Hugo Bellen
Richard Benton
Lisa Bero
Avinash Bhandoola
Russell Bonduriansky
Isabelle Boutron
Joshua Brickman
James Briscoe
Marianne Bronner
Kenneth Cadwell
Heather Cameron
Judith Campisi
Fernanda Ceriani
Xuemei Chen
Lars Chittka
Heather Christofk
Isabelle Côté
Matthew Dalva
Jeffery Dangl
Frans de Waal
Ghislaine Dehaene-Lambertz
Claude Desplan
Ulrich Dirnagl
Andrew Dobson
Michael Doebeli
Xinnian Dong
Daniel Durocher
Raimund Dutzler
Connie Eaves
Bruce Edgar
Jonathan Eisen
Mark Estelle
Christophe Fraser
Susan Gasser
Jeff Gore
Alex Gould
Douglas Green
Ueli Grossniklaus
Hiroshi Hamada
Stacey Harmer
William A. Harris
Bassem Hassan
Anders Hedenström
Joseph Heitman
Daniel Herschlag
Caroline Hill
David Hillis
Sui Huang
Simon Hughes
Frederick Hughson
Laurence Hurst
Anna Huttenlocher
Nancy Hynes
Robert Insall
John Ioannidis
Elisa Izaurralde
Ole Jensen
Gerald Joyce
James Kadonaga
Sophien Kamoun
Laurent Keller
Cheryl Kerfeld
Chaitan Khosla
Jonathan Kimmelman
Thomas Kirkwood
Katia Koelle
Adam Kohn
Achim Kramer
Nina Kraus
Arthur Lander
Matthias Landgraf
Michael Laub
Susan Lindquist
Cecilia Lo
Michel Loreau
Sally Lowell

Georgina Mace
Laura Machesky
Malcolm Macleod
Harmit Malik
Philippa Marrack
Sophie Martin
Bénédicte Michel
Tom Misteli
Aaron Mitchell
Nancy Moran
Craig Moritz
Hélène Morlon
Leonie Moyle
Mary Mullins
June Nasrallah
David Nemazee
Eric Nestler
Phillip Newmark
Roel Nusse
Christopher Pack
Carole Parent
David Pellman
David Penny
Gregory Petsko
Jonathon Pines
Hidde Ploegh
David Poeppel
Franck Polleux
Sara Rankin
Andrew Read
Felix Rey
Jeremy Rich
Steven Riley
David Ron
Sarah Rowland-Jones
Matthew Rushworth
Peter Scheiffele
Sandr Schmid
David Schneider
Timm Schroeder
Trina Schroer
Maya Schuldiner
Idan Segev
Piali Sengupta
Mark Siegal
Daniel Simberloff
Anne Simonsen
Agata Smogorzewska
Derek Stemple
Charles F. Stevens
Ann Stock
Kate Storey
Boris Striepen
Bill Sugden
Paul Taghert
Nicolas Tapon
Graham Taylor
Janet Thornton
Rong Tian
Heidi Tissenbaum
Frank Tong
Christopher Tyler-Smith
Leslie Ungerleider
Matt van de Rijn
David Vaux
Antonio Vidal-Puig
Leslie Vosshall
Eric-Jan Wagenmakers
Matt Waldor
Peter Walter
Gary Ward
Jonathan Weissman
Mariana Wolfner
Yukiko Yamashita
Phillip Zamore
Robert Zatorre
