# Supplementary material for: PLOS Biology 2016 Reviewer and Editorial Board Thank You
Source: PLoS Biol. 2017 Mar 20;15(3):e2002409. doi: 10.1371/journal.pbio.2002409 (PMC5358729; doi:10.1371/journal.pbio.2002409)
Supplement: S1 Guest Editor List — (PDF) [file pbio.2002409.s003.pdf]

PLOS Biology would like to thank all those who served as Guest Academic Editors in 2016:

Asa Abeliovich
Cliff Abraham
Martin Ackermann
Ralph Adolphs
Juan Alfonzo
Stephen Baccus
Francesco Battaglia
Craig Blackmore
Craig Blackstone
Patrice Bourgin
Simon Bullock
Vern Carruthers
BJ Casey
Jamie Cate
Yury Chernoff
Leonardo Cohen
Barbara Conradt
Pierre-Jean Corringer
Jozsef Csicsvari
Nathaniel Daw
Arjan de Visser
Michael Dickinson
Jonathan Dinman
Joshua Dubnau
Maitreya Dunham
Timothy Ebner
Stephen Ellner
Michael Emerman
Ben Emery
Andreas Engel
Grigori Enikolopov
Cagla Eroglu
Michael Fanselow
Charles ffrench-Constant
Kevin Foster
Michael Frank
Angela Friederici
Karl Friston
Fred Gage
Karunesh Ganguly
Timothy Gardner
Holger Gerhardt
Jennifer Gerton
Asif Ghazanfar
Robert Gifford
Wendy Gilbert
Margaret Goodell
Anthony Graham
Michael Granato
Jeremy Green
Bryan Grenfell
Eric Haag
Eddie Holmes
Michael Johansson
Gareth John
Pedro Jordano
Natalia Jura
Philipp Khaitovich
James Kilner
A. Marm Kilpatrick
Carla Kim
David Kleinfeld
Hanna Kokko
Genevieve Konopka
Nick Lane
Nicholas Levinson
Jason Locasale
Matthieu Louis
Claire Marris
Susan McCouch
Imelda McGonnell
Frauke Melchior
Elliot Meyerowitz
Edward Mitre
Denise Monack
Lisa Monteggia
Walther Mothes
Coleen Murphy
Maiken Nedergaard
Thomas Nutman
Howard Ochman
Mary O'Riordan
Carl Petersen
Dietmar Plenz
Michael Posner
Emma Rawlins
Jason Reed
Roland Regoes
David Relman
Kerry Ressler

Robert Ricklefs
Carol Robinson
Eduardo Rocha
Michael Rout
Michael Schatz
Erin Schuman
Thomas Schwartz
Ben Seymour
Celeste Simon
Michael Simons
Mikael Simons
Michael Sixt
Hongjun Song
Victor Sourjik
Sarah Teichmann
Olivier Tenaillon
Joe Thornton
Sharon Tooze
Frank Uhlmann
James Umen
Alexander van Oudenaarden
Tor Wager
Vincent Walsh
Brian Wandell
Michael Whitfield
Daniel Wolpert
Wayne Yokoyama
Yossi Yovel
Anthony Zador
Hongkui Zeng
